# H_2_O-Induced Hydrophobic Interactions in MS-Guided Counter-Current Chromatography Separation of Anti-Cancer Mollugin from *Rubia cordifolia*

**DOI:** 10.3390/molecules26030751

**Published:** 2021-02-02

**Authors:** Liping Zeng, Tianyi Xu, Jie Meng, Dingfang Wu, Shihua Wu

**Affiliations:** 1 Department of Thoracic Surgery, First Affiliated Hospital, College of Medicine, Zhejiang University, Hangzhou 310003, China; zheda1987@163.com; 2 Research Center of Siyuan Natural Pharmacy and Biotoxicology, College of Life Sciences, Zhejiang University, Hangzhou 310058, China; 21807009@zju.edu.cn (T.X.); 18758021881@163.com (J.M.); wudingfang0725@163.com (D.W.); 3 College of Resource and Environment, Qingdao Agricultural University, Qingdao 266109, China; 4 Rui’an Food Inspection and Testing Center, Rui’an 325204, China; 5 Joint Research Centre for Engineering Biology, Zhejiang University—University of Edinburgh Institute, Zhejiang University, Haining 314400, China

**Keywords:** counter-current chromatography, hydrophobic interactions, mollugin, natural products isolation, *Rubia cordifolia*, traditional Chinese medicine

## Abstract

Counter-current chromatography (CCC) is a unique liquid–liquid partition chromatography and largely relies on the partition interactions of solutes and solvents in two-phase solvents. Usually, the two-phase solvents used in CCC include a lipophilic organic phase and a hydrophilic aqueous phase. Although a large number of partition interactions have been found and used in the CCC separations, there are few studies that address the role of water on solvents and solutes in the two-phase partition. In this study, we presented a new insight that H_2_O (water) might be an efficient and sensible hydrophobic agent in the *n*-hexane-methanol-based two-phase partition and CCC separation of lipophilic compounds, i.e., anti-cancer component mollugin from *Rubia cordifolia*. Although the *n*-hexane-methanol-based four components solvent systems of *n*-hexane-ethyl acetate-methanol-water (HEMWat) is one of the most popular CCC solvent systems and widely used for natural products isolation, this is an interesting trial to investigate the water roles in the two-phase solutions. In addition, as an example, the bioactive component mollugin was targeted, separated, and purified by MS-guided CCC with hexane-methanol and minor water as a hydrophobic agent. It might be useful for isolation and purification of lipophilic mollugin and other bioactive compounds complex natural products and traditional Chinese medicines.

## 1. Introduction

Counter-current chromatography (CCC) is a versatile liquid-liquid partition chromatography separation technique without solid support-matrix [1]. It largely relies on the partition interactions of solutes and solvents in the two-phase solvent systems. Usually, the selection of a two-phase solvent system is the first and also the most important step for the CCC separation process [2]. So far, a large number of two-phase solvent systems [3,4,5] have been explored to separate many kinds of compounds such as from natural products [3,6,7,8] and traditional Chinese medicines (TCMs) [9]. Among these solvent systems used for the CCC process, the system composed of *n*-hexane-ethyl acetate-methanol-water (HEMWat) may be the most popular solvent system for the counter-current isolation of complex natural products ranging from lipophilic to hydrophilic components [6,10,11,12]. Previous literature survey [6] indicated that about 29% of studies employ HEMWat combination solvents to perform the featured liquid-liquid separation since 1982. The advantages of using HEMWat combination solvent systems are that short settling time, high retention of stationary phase, and well peak resolution might be easily achieved on the current CCC apparatus including type-I, J, and axis-cross devices from analytical scale analysis to preparative isolation and even to industrial preparation [13,14,15,16,17,18,19,20]. In addition, there are some significant linear tendencies between the phase composition of the solvent systems and the log of solute distribution constants, and thus it is relatively easy to obtain appropriate partition coefficients (*K*) suitable for CCC separation by simply changing the relative proportions [21]. Furthermore, using extended HEMWat 9 × 9 map systems, it is possible to obtain an infinite number of HEMWat systems with the same partition coefficients [12]. Therefore, these HEMWat solvent systems were widely accepted as the first solvent candidates for the selection of CCC solvents [2,6,10,12,21].

**Figure 1:**
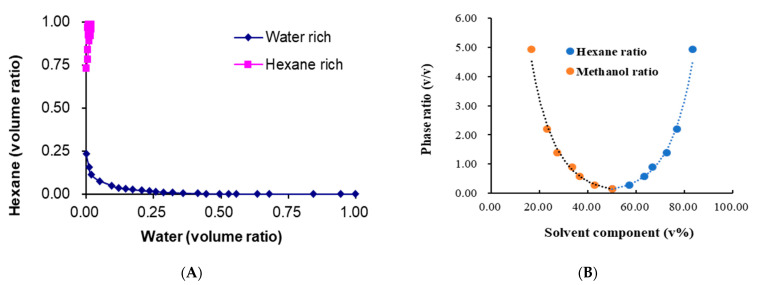
(**A**) The phase diagram analysis of *n*-hexane-methanol-water system and (**B**) phase volume ratio of non-aqueous *n*-hexane-methanol. Liquid-liquid equilibria data were adopted from [22] and validated in our lab.

In general, the four components of the HEMWat solvent family or maps are originated from the simplest two-phase solvent composed of *n*-hexane and methanol, which is a very interesting two-phase solvent system [22], and its property is very similar to its analog combination of heptane-methanol-water [23]. As shown in Figure 1A, if the content of hexane ranges from 25% to 75% in the whole volume, non-aqueous hexane-methanol two-phases could be stably formed. However, when all ternary compositions with more than 1% *v*/*v* water, two liquid phases form, one of which is practically pure hexane (or heptane) [23]. Although its liquid–liquid equilibria have been well known (Figure 1A) [22], they are still limited to knowing partition interactions between solvents and solutes.

**Figure 2:**
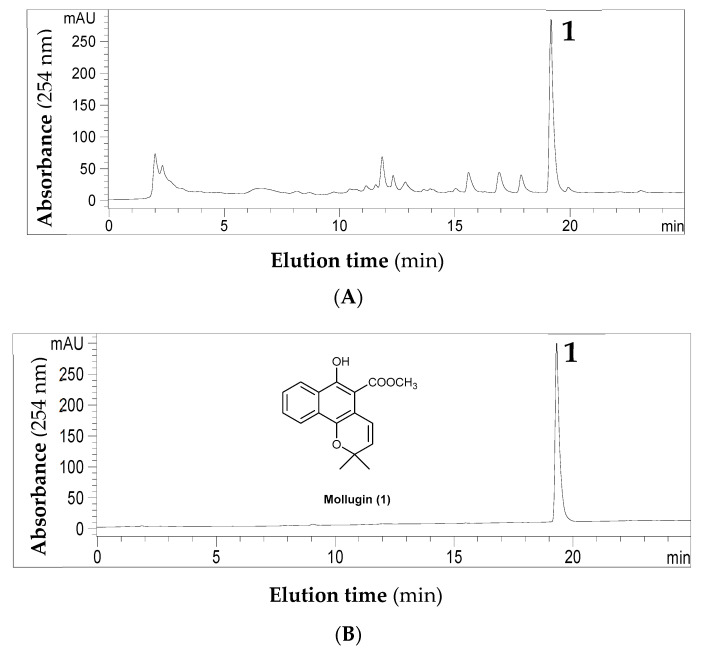
The representative HPLC analyses of (**A**) crude ethyl acetate extract from *R. cordifolia* and (**B**) the product purified by counter-current chromatography (CCC). **1**, mollugin. HPLC conditions: C18 column (ZORBAX Eclipse XDB-C18, 150 mm length × 4.6 mm I.D., 5 μm); mobile phase: solvent M (methanol) and W (water) in the gradient mode as follows: 0–20 min, 60–100% M; 20–25 min, 100% M; the flow rate was 0.8 mL min^−1^.

Usually, water is called the universal solvent because it can dissolve more substances than any other liquid. In CCC separation, water is also a common solvent to compose the aqueous phase, while other lipophilic organic solvents form the non-aqueous phase. For example, in HEMWat systems, water is a major component to determine the polarity of whole two-phase solvents. A higher ratio of water in the two-phase system means having more content of water in the aqueous phase. Thus, in these multiple components solvent systems, water has a significant hydrophilic effecton the polar solutes and solvents, resulting in more ethyl acetate, methanol, and solutes in the aqueous phase and forming lower *K* values [12,21]. For binary systems of *n*-hexane-methanol, it is not appropriate for the separation of polar compounds but suitable for non-polar or lipophilic components. Using a lipophilic mollugin (**1**) (Figure 2) [24,25,26,27], a major anti-cancer component of *Rubia cordifolia* as a target sample, we found that water was a very efficient and sensible hydrophobic agent in the mollugin-solved *n*-hexane-methanol solvent systems. Minor water could significantly improve the retention of stationary phase and partition eco-efficiencies of mollugin.

Therefore, in this study, we aim to give a short report on the hydrophobic role of water in the partition and practical separation of mollugin using *n*-hexane-methanol two-phase solvent systems. Although water plays possibly versatile roles in the two-phase partition and CCC separation [21], this is a useful trial to apply the hydrophobic role of water to separate the lipophilic mollugin. In addition, to the best of our knowledge, this is the first document to investigate the *n*-hexane-methanol-water ternary solvent system for isolation of mollugin although there are several CCC methods to separate mollugin from the roots of *R. cordifolia*, using a multiple-components two-phase solvent system composed of light petroleum-ethanol-diethyl ether-water (5:4:3:1 *v*/*v*) [28,29]. Comparisons with the multiple-components solvents, the binary *n*-hexane-methanol, or ternary *n*-hexane-methanol-water solvent systems are easier to recycle and regenerate into single solvent by simple solvent purification such as flash distillation or other methods. It is useful for the separation of mollugin from the roots of *R. cordifolia* and might be applied for the separation of other components from complex natural products.

## 2. Results and Discussion

### 2.1. HPLC Analyses of Crude Extracts and Purified Targets

As shown in Figure 2, the crude ethyl acetate extract of *R. cordifolia* was first analyzed by HPLC-DAD-ESI-MS. Although there were many components in the extract, the selective negative molecular ion at *m*/*z* 283 [M − H]^−^ indicated that peak 1 was a possible target mollugin, which was confirmed further by NMR after CCC purification. HPLC results also indicated that primary extraction by ethyl acetate-water is efficient for the enrichment of mollugin from the complex crude ethanol extracts. After ethyl acetate-water extraction, the content of the mollugin was reached about 20%, which was used as a sample for CCC separation.

### 2.2. H_2_O-Induced Significant Hydrophobic Roles both on the Solvents and Target

As described above, *n*-hexane-methanol-water is an interesting ternary solvent system. As shown in Figure 1A, the ternary *n*-hexane-methanol-water solvent system has wide two-phase layered zones and can form aqueous and non-aqueous hexane-methanol two-phase systems. When the volume ratio of hexane reaches more than 25% or volume ratio of methanol is less than 75%, both the two-components of hexane-methanol system and three-components of hexane-methanol-water system can form the layered two phases. The non-aqueous, hexane-methanol two-phase system may be formed in the zones of that volume ratio of hexane is more than 25% and methanol is less than 75%. For efficient CCC isolation, the ratio of two phases is usually required to be more than 0.50, and thus the optimum volume ratio for non-aqueous CCC separation is more than 60% for hexane and less than 40% for methanol. It should be noted that with the increasing of hexane volume ratio, the volume ratio of the formed two phases increases exponentially (Figure 1B). However, the partition coefficient of the hydrophobic mollugin made almost no changes (Table 1). This may be due to the minor component changes of the upper and lower phases even though their phase volumes were changed sharply. In addition, the non-aqueous hexane-methanol system could not be used for continuous repeat preparation using the same stationery because the stationary phase accompanied to be eluted little by little after dynamic-equilibrium during the CCC process, which may be due to the minor mass difference between two phases although they may be settled soon. At the end of the CCC process (data are not shown), the final retention of the stationary phase was little (less than 30%) by comparison with the first retention on the dynamic equilibrium (more than 50% retention).

However, *n*-hexane-methanol two-phase solvent systems were found to be very sensitive to water similar to the ternary heptane-water-methanol system [23]. For example, the stable two phases with short settling time may be formed only by adding a very minor content of water into the above hexane-methanol non-aqueous systems. In addition, the partition coefficients of targets will change sharply with the addition of water. Only by adding about 1% water, the stable retention of the stationary phase could be obtained. Therefore, water might be an important additive for *n*-hexane-methanol solvents, and worth investigating and applying in the separation of mollugin.

**Figure 3:**
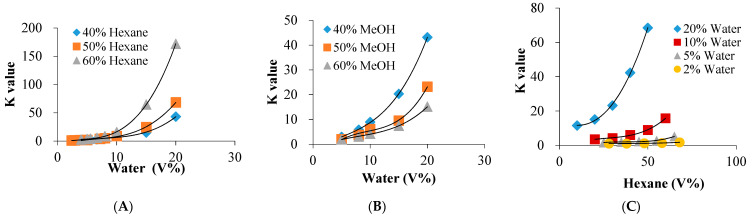
The relationship of partition coefficients of mollugin with the changes of solvent components. There were increase trends of *K* values under keeping different contents of (**A**) hexane, (**B**) methanol and (**C**) water.

As shown in Figure 3A and Table S1, water showed a significant hydrophobic role to drive hexane and mollugin into hexane-rich upper phase, yielded in increasing phase volume ratio and larger *K* values with increasing the content of water. This was confirmed further by experiments of decreasing the methanol content. As shown in Figure 3B, and Table S2, the system with a lower ratio of methanol possessed higher upper phase and higher *K* values, and higher content of methanol produced smaller *K* values for mollugin. Furthermore, as shown in Figure 3C and Table S3, higher content of water means larger *K* values. This water-produced hydrophobic role on hexane and mollugin may be useful for CCC separation of lipophilic compounds.

### 2.3. Preparative Aqueous CCC Isolation of Mollugin

It is well known that the successful CCC separation still largely depends on the selection of two-phase solvent systems. Generally speaking, two key factors should be considered before CCC separation [21]. One is to compose a solvent system with a good two-phase separation, which results from the solvent-solvent interaction with/without additives. The used solvent system has clear two liquid phases (upper light phase and lower heavy phase) and short settling time (phase separation time). Usually, the settling time of two phases was less than 30 s required for a successful type-J CCC separation. The other factor is to make the solutes have appropriate partition coefficients (*K*) in the solvent systems, which depends on the whole interaction between two phases and solutes (targets). The suitable *K* values for high-speed CCC are in the range of 0.5 ≤ *K* ≤ 1 [2], and the “sweet spot” is centered at *K* = 1, while 0.4 < *K* < 2.5 represents a conservative range [11]. In the extrusion mode such as elution-extrusion CCC [30], the “sweet spot” can be significantly extended toward higher *K* ranges (0.25 < *K* < 16) [31].

Therefore, according to the above experimental results, the acceptable solvent systems were that the volume ratio of water was controlled less than 5% and the content of hexane or methanol was about 50%. As shown in Table 2, several ternary solvent systems were selected as two-phase solvents for CCC separation and yielded high and stable retention of stationary phase (more than 50%) and high pure mollugin fractions (more than 97% purity).

**Figure 4:**
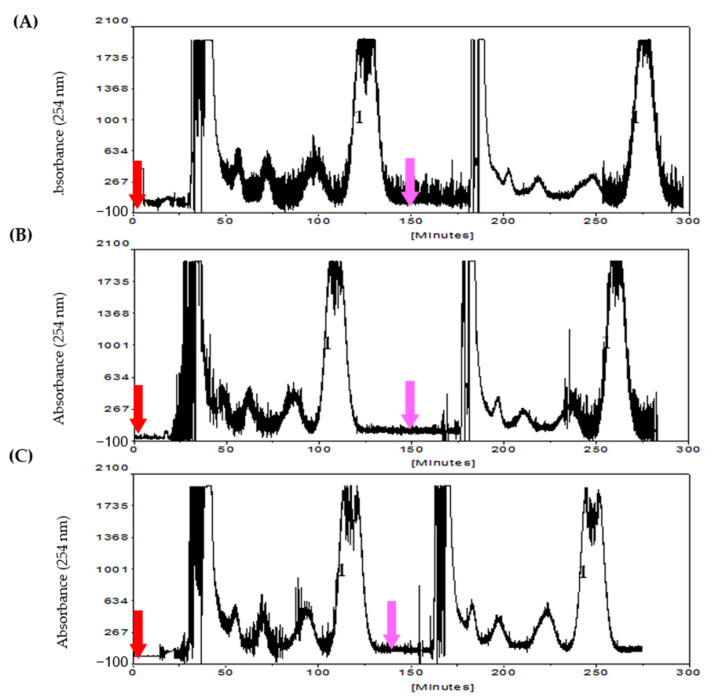
Representative profiles of preparative CCC isolation of mollugin using aqueous hexane-methanol systems with two continuous injections; **1**, mollugin. Solvent systems: (**A**) 13:16:1, (**B**) 14:18:1, and (**C**) 13:20:1. Red arrow, the first sample injection; pink arrow, the second sample injection. Sample size, 100 mg/injection; flow rate, 3.5 mL/min; detections at 254 nm.

Typical CCC isolations are illustrated in Figure 4. Using the systems composed of hexane-methanol-water (13:16:1, 14:18:1, 13:20:1, or in percent 43.3:53.3:3.3, 42.4:54.6:3.0, 38.2:58.8:3.0, *v*/*v*), the mollugin could be isolated in more than 97% purity within 150 min in a single CCC run. The products purified by CCC were determined by HPLC and further validated by NMR spectra. Representative NMR spectra are listed in Figure S1, which further confirmed the purity of the product purified by CCC. In addition, because the stationary phase was well retained in the CCC column, the multiple repeat injections could be performed using the same stationary phase after the elution of the target in the first CCC run. Figure 4 clearly indicates that these are not significant effects on CCC isolation using the stationary phase of the last CCC run. By comparison with the non-aqueous *n*-hexane-methanol solvents, aqueous *n*-hexane-methanol solvent system (minor water, less than 5%) demonstrated more suitable for lipophilic mollugin because of more stable retention of stationary phase and higher sample capacity (Table 2).

## 3. Materials and Methods

### 3.1. Chemical and Reagents

All organic solvents used for CCC purification were of analytical grade and purchased from Huadong Chemicals, Hangzhou, China. The water was purified by means of a water purifier (18.2 MΩ) (Wanjie Water Treatment Equipment Co., Ltd., Hangzhou, China) and used for all solutions and dilutions. Methanol used for HPLC analysis was of chromatographic grade and purchased from Merck, Darmstadt, Germany.

The dried roots of *R. cordifolia* were purchased from Huqingyutang Museum of Traditional Chinese Medicines (Hangzhou, China) and identified by the Institute of plant science, Zhejiang University, Hangzhou, China.

### 3.2. Apparatus and Agents

The CCC instrument employed in the present study is a TBE-300A high-speed CCC (Tauto Biotech. Co., Ltd., Shanghai, China) with three multilayer coil separation columns connected in series (i.d. of the tubing, 1.8 mm; total column volume, 260 mL, and extra volume, 10 mL) and a 20 mL sample loop. The revolution radius was 5 cm, and the β values of the multilayer coil varied from 0.5 at the internal terminal to 0.8 at the external terminal. The revolution speed of the apparatus can be regulated with a speed controller in the range between 0 rpm and 1000 rpm. A TC 1050 constant-temperature circulating instrument (Tauto Biotech. Co., Ltd., Shanghai, China) was used to control the separation temperature. In addition, this CCC system is equipped with a P270 metering pump, a UV 230+ spectrometer (Elite Analytical Instrument Co., Ltd., Dalian, China), BSZ-100 fraction collector, and EC2000 ChemStation (Elite Analytical Instrument Co., Ltd., Dalian, China).

The high-performance liquid chromatography (HPLC) used was an Agilent 1100 system (Agilient Technologies Inc., Santa Clara, CA, USA) including a G1379A degasser, a G1311A QuatPump, a G1367A Wpals, a G1316A column oven, a G1315B diode assay detector (DAD), and an Agilent ChemStation for LC.

### 3.3. Preparation of the Crude Extract

The dried roots of *R. cordifolia*, family *Rubiaceae,* were ground into powder. A total of 500 g of the powder was extracted three times with 2 L of 80% ethanol for 2 h under reflux for three times (a total volume of 6 L). The extracts were combined, evaporated to dryness under reduced pressure at 45 °C. The crude extract was re-dissolved in 1 L water, again extracted with ethyl acetate (1 L × 3 times). The ethyl acetate extracted solutions were combined and evaporated to dryness to yield 6.97 g of ethyl acetate extract as the crude sample.

### 3.4. Measurement of Two-Phase Volume Ratio and Partition Coefficient (K)

Based on the data of the phase diagram of the hexane-methano-water system (Figure 1), some solvent systems (total 10 mL) with desired volume ratio were prepared in the sealable 15 mL glass tubes. After being shaken for 2 min on a vortex mixer, the newly formed two-phase were separated and the volumes of the upper and lower phases were measured accurately. The two phases volume ratio was calculated as the volume of the upper phase/the volume of the lower phase.

The partition coefficients (*K*) for the target compound were determined by HPLC analysis as follows: a small amount (1 mg) of the crude sample was dissolved into equal volume (800 μL) of aqueous phase (lower phase) and organic phase (upper phase) of the thoroughly equilibrated two-phase solvent system in a 2 mL test tube. After the equilibration was established, both the upper phase and the lower phase were analyzed by HPLC, and the peak area of the target in the upper phase and in the lower phase was recorded as *A*_1_ and *A*_2_, respectively. The partition coefficient (*K*) was then calculated by the following equation *K* = *A*_1_/*A*_2_. *K* value of each two-phase solvent was tested three times at least, and their average value was adopted in tables.

### 3.5. Preparation of Two-Phase Solvent Systems and Sample Solution

After schematic tests of *K* values by HPLC, the appropriate solvent systems composed of *n*-hexane-methanol were selected and prepared. The solvent mixture was thoroughly equilibrated in a separatory funnel at room temperature and the two phases separated shortly before use.

The sample solution for further CCC purification was prepared by dissolving the crude sample in the lower phase (20 mL).

### 3.6. MS-Guided Preparative CCC Separation Procedure

Although the direct online elstrospray ionization mass spectrometry(ESI-MS) analysis [32,33] might rapidly detect the target component fractionalized by the coupling CCC, due to high cost, here we still employed offline ESI-MS guided CCC separation for targeted isolation of the bioactive mollugin [34,35]. The CCC column was first entirely filled with the upper phase of the solvent system as stationary phase and then about 20 mL of the sample solution was injected through the injection valve. The lower phase as mobile phase pumped through the column from head to tail and the temperature was set at 30 °C. The effluent was monitored with a UV detector at 254 nm and automatically collected in a 20 mL test tube using a BSZ-100 fraction collector. Peak fraction was collected according to the elution profile. After the targeted peak had been eluted, the CCC run was stopped and the residual stationary phase retained in the column was pushed out to calculate retention of the stationary phase. For the non-aqueous CCC isolation, the rotary speed of the CCC device was controlled at 800 rpm and the flow rate was set at 1–1.5 mL min^−1^ because some upper phase was easily co-eluted with lower phase. In contrast, for the aqueous CCC isolation, the rotary speed was set at 900 rpm, and the flow rate was 3.5 mL min^−1^ due to the stable retention of the stationary phase.

### 3.7. HPLC Analysis and Identification of CCC Peak Fraction

The HPLC analysis was performed on a C18 column (ZORBAX Eclipse XDB-C18, 150 mm length × 4.6 mm I.D., 5 μm, purchased from Agilient Technologies Inc. The mobile phase was a linear gradient of methanol (M) and water (W) as follows: 0–20 min, 60–100% M; 20–25 min, 100% M. The flow rate was 0.8 mL min^−1^, and detection wavelength was set at 254 nm. Other conditions included column temperature, 25 °C; and injection volume, 10 μL. The purity analyses were tested three times at least and their average purities were adopted in tables.

Identification of the CCC fraction was carried out by ESI-MS and NMR spectra. Negative ESI-MS analyses were performed with a Thermo Fignnigan LCQ Deca XP ESI_MS (Thermo Electron Corporation, Waltham, MA, USA). NMR experiments were performed on a Bruker Advance DMX 500 NMR spectrometer (The Bruker Corporation, Billerica, MA, USA) with chloroform (CDCl_3_) as solvent and tetramethylsilane (TMS) as internal standard. The compound 1 obtained by CCC showed the characteristic ion at *m*/*z* 283 due to [M − H]^−^, corresponding to molecular weight of 284 of mollugin. ^1^H NMR data (500 MHz, CDCl_3_): δ = 1.49 (6H, s, -CH_3_), 4.02 (3H, s, -OCH_3_), 5.67 (1H, d, *J* = 10.0 Hz, H-2′), 7.11 (1H, d, *J* = 10.0 Hz, H-1′), 7.51–7.62 (2H, m, H-6, 7), 8.17 (1H, dd, *J* = 8.3 Hz, H-8), 8.36 (1H, dd, *J* = 8.3 Hz, H-5), 12.17 (1H, s, ArOH). ^13^C NMR data (125 MHz, CDCl_3_): δ = 172.51 (-C=O), 156.50 (C-1), 141.57 (C-4), 129.34 (C-6), 129.0 (C-4a), 128.86 (C-2′), 126.3 (C-7), 125.07 (C-8a), 124.02 (C-5), 122.3 (C-1′), 121.92 (C-8), 112.56 (C-3), 102.21 (C-2), 74.65 (C-3′), 52.32 (-OCH3), 26.85 (C-4′), 26.85(C-5′). These data were identical to the previously published studies [28,29,36].

## 4. Conclusions

There are several kinds of hydrophilic and hydrophobic interactions between solvents and solutes to be found and used in the CCC separation. However, there are still relatively unknown interactions in the CCC practice. In this study, we revisited the classical hexane-methanol aqueous and non-aqueous solvent systems for the isolation of lipophilic mollugin. We found that water might play an important hydrophobic role in the solvent hexane-methanol for the partition of mollugin. By adding minor ratio water (less 5%), the retention of stationary phase and the partition eco-efficiencies of mollugin can be significantly improved and several successful CCC separations have been performed. Moreover, the binary *n*-hexane–methanol or ternary *n*-hexane-methanol-water solvent systems are easier to recycle than other multiple-component, two-phase solvent systems.

Although the solvent systems have been widely used for CCC separation, and the target mollugin has been isolated by other methods, this study provided new insights and a simple separation strategy by using hexane-methanol with minor water. It might be useful for the isolation of lipophilic mollugin and other bioactive compounds.

## Figures and Tables

**Table 1:** The solvent components and *K* values.

Solvent System ^a^	System Components (v %)		Phase Volume Ratio	*K* ^b^
(Hexane:Methanol, *v*/*v*)	Hexane	Methanol	V_U_/V_L_	1
3:3	50.00	50.00	0.16	0.85
4:3	57.14	42.86	0.28	0.83
1.9:1.1	63.33	36.67	0.58	0.80
6:3	66.67	33.33	0.81	0.82
8:3	72.73	27.27	1.39	0.79
10:3	76.92	23.08	2.20	0.79
15:3	83.33	16.67	4.94	0.78

^a^*n*-Hexane-methanol. ^b^*K* value was expressed as the peak area of the compound in the upper phase divided by the peak area of the compound in the lower phase.

**Table 2:** Typical solvents for preparative isolation of mollugin by CCC.

Solvent System			Solvent Components (v %)			Phase Ratio	*K* ^a^	Rf ^b^ (%)	T_R_/min	Duration (min)	Used Lower Phase (mL)	Purity by HPLC (%)
Hexane	MeOH	Water	*n*-Hexane	Methanol	Water							
9	10	1	45.00	50.00	5.00	0.67	2.63	66.67	188	208	624	97.32
10	11	1	45.45	50.00	4.55	0.76	2.36	65.19	170	199	597	97.41
10	12	1	43.48	52.17	4.35	0.61	2.23	66.30	140	157	550	97.27
13	15	1	44.83	51.72	3.45	0.61	2.12	64.81	133	147	515	98.43
12	14	1	44.44	51.85	3.70	0.63	1.92	63.33	122	146	511	98.42
13	16	1	43.33	53.33	3.33	0.55	1.94	59.26	120	140	490	97.96
14	18	1	42.42	54.55	3.03	0.51	1.65	55.55	105	121	424	99.08
13	20	1	38.24	58.82	2.94	0.40	1.79	55.55	111	131	459	97.10
15	20	1	41.67	55.56	2.78	0.47	1.61	61.11	106	127	445	97.38
17	20	1	44.74	52.63	2.63	0.57	1.69	54.44	106	120	420	97.24

^a^ Measured by CCC experimental results. ^b^ Rf, the retention of stationary phase. Due to the presence of water, the stationary phase was retained stably in the column and did not continue to flow out the CCC column eluted with mobile phase after dynamic equilibrium.

## Data Availability

All the data associated with present manuscript have been included in supplementary materials.

## Supplementary Materials

The following are available online, Figure S1: The representative ^1^H and ^13^C NMR spectra of mollugin isolated by CCC with the two-phase solvents of *n*-hexane-methanol-water, Table S1: The *K* values and phase volume ratio of mollugin in several solvent systems, Table S2: The *K* values and phase volume ratio of mollugin in several solvent systems, Table S3: The *K* values and phase volume ratio of mollugin in several solvent systems.

## Sample Availability

Samples of the compounds are not available from the authors.
