# Supplementary material for: H2O-Induced Hydrophobic Interactions in MS-Guided Counter-Current Chromatography Separation of Anti-Cancer Mollugin from Rubia cordifolia
Source: Molecules. 2021 Feb 2;26(3):751. doi: 10.3390/molecules26030751 (PMC7867130; doi:10.3390/molecules26030751)
Supplement: Supplementary file 1 [file molecules-26-00751-s001.pdf]

Supplementary Materials

H_2O-Induced Hydrophobic Interactions in MS-Guided Counter-Current Chromatography Separation of Anti-Cancer Mollugin from *Rubia cordifolia*

Liping Zeng [1,2], Tianyi Xu [2], Jie Meng [2,3], Dingfang Wu [2,4] and Shihua Wu [2,5,*]

[1] Department of Thoracic Surgery, First Affiliated Hospital, College of Medicine, Zhejiang University, Hangzhou, Zhejiang 310003, China; zheda1987@163.com
[2] Research Center of Siyuan Natural Pharmacy and Biotoxicology, College of Life Sciences, Zhejiang University, Hangzhou, Zhejiang 310058, China; 21807009@zju.edu.cn (T.X.); 18758021881@163.com (J.M.); wudingfang0725@163.com (D.W.)
[3] College of Resource and Environment, Qingdao Agricultural University, Qingdao, Shandong 266109, China
[4] Rui'an Food Inspection and Testing Center, Rui'an, Zhejiang 325204, China
[5] Joint Research Centre for Engineering Biology, Zhejiang University-University of Edinburgh Institute, Zhejiang University, Haining 314400, China
* Correspondence: drwushihua@zju.edu.cn; Tel./Fax: +86-571-88206287

Table S1. The *K* values and phase volume ratio of mollugin in several solvent systems[a]

Solvent system (v%)			Phase volume ratio	K^b
***n*-Hexane**	**Methanol**	**Water**	**V_U/V_L**	**A_U/A_L**
40.00	40.00	20.00	0.68	43.16
40.00	45.00	15.00	0.68	15.18
40.00	50.00	10.00	0.63	8.22
40.00	52.00	8.00	0.60	4.31
40.00	53.33	6.67	0.57	3.26
40.00	54.44	5.56	0.55	2.37
40.00	55.00	5.00	0.52	2.05
40.00	56.00	4.00	0.48	1.71
50.00	30.00	20.00	1.04	68.45
50.00	35.00	15.00	1.03	24.71
50.00	40.00	10.00	0.99	8.95
50.00	42.00	8.00	0.97	5.09
50.00	42.86	7.14	0.91	3.94
50.00	45.00	5.00	0.87	2.00
50.00	46.15	3.85	0.82	1.95
50.00	47.06	2.94	0.75	1.40
50.00	47.62	2.38	0.73	1.10
60.00	20.00	20.00	1.55	172.11
60.00	25.00	15.00	1.55	64.76
60.00	30.00	10.00	1.55	15.78
60.00	32.00	8.00	1.55	8.74
60.00	33.33	6.67	1.43	5.89
60.00	34.44	5.56	1.43	4.32
60.00	35.00	5.00	1.45	3.62
60.00	36.00	4.00	1.36	2.82

[a] *n*-Hexane-methanol-water.

[b] *K* value was expressed as the peak area of the compound in the upper phase divided by the peak area of the compound in the lower phase.

Table S2. The *K* values and phase volume ratio of mollugin in several solvent systems[a]

Solvent system (v%)			Phase volume ratio	K[b]
***n*-Hexane**	**Methanol**	**Water**	V_U/V_L	A_U/A_L
40.00	40.00	20.00	0.68	43.16
45.00	40.00	15.00	0.81	20.31
50.00	40.00	10.00	0.97	8.95
52.00	40.00	8.00	1.02	5.85
55.00	40.00	5.00	1.12	2.97
30.00	50.00	20.00	0.43	23.20
35.00	50.00	15.00	0.53	9.52
40.00	50.00	10.00	0.63	6.08
42.00	50.00	8.00	0.66	3.69
45.00	50.00	5.00	0.70	2.00
20.00	60.00	20.00	0.23	15.11
25.00	60.00	15.00	0.31	7.39
30.00	60.00	10.00	0.35	4.16
32.00	60.00	8.00	0.37	3.07
35.00	60.00	5.00	0.38	1.88

[a] *n*-Hexane-methanol-water.

[b] *K* value was expressed as the peak area of the compound in the upper phase divided by the peak area of the compound in the lower phase.

Table S3. The *K* values and phase volume ratio of mollugin in several solvent systems[a]

Solvent system (v%)			Phase volume ratio	*K*[b]
n-Hexane	Methanol	Water	V_U/V_L	A_U/A_L
50.00	30.00	20.00	1.04	68.45
40.00	40.00	20.00	0.68	42.29
30.00	50.00	20.00	0.43	23.20
20.00	60.00	20.00	0.23	15.10
10.00	70.00	20.00	0.08	11.54
60.00	30.00	10.00	1.55	15.78
50.00	40.00	10.00	0.99	8.95
40.00	50.00	10.00	0.63	6.08
30.00	60.00	10.00	0.35	4.26
20.00	70.00	10.00	0.18	3.55
65.00	30.00	5.00	1.44	5.19
55.00	40.00	5.00	1.13	2.78
45.00	50.00	5.00	0.68	2.30
35.00	60.00	5.00	0.38	1.88
25.00	70.00	5.00	0.17	1.67
68.00	30.00	2.00	1.86	1.77
58.00	40.00	2.00	1.06	1.34
48.00	50.00	2.00	0.60	1.23
38.00	60.00	2.00	0.27	1.04
28.00	70.00	2.00	0.06	1.01

[a] *n*-Hexane-methanol-water.

[b] *K* value was expressed as the peak area of the compound in the upper phase divided by the peak area of the compound in the lower phase.

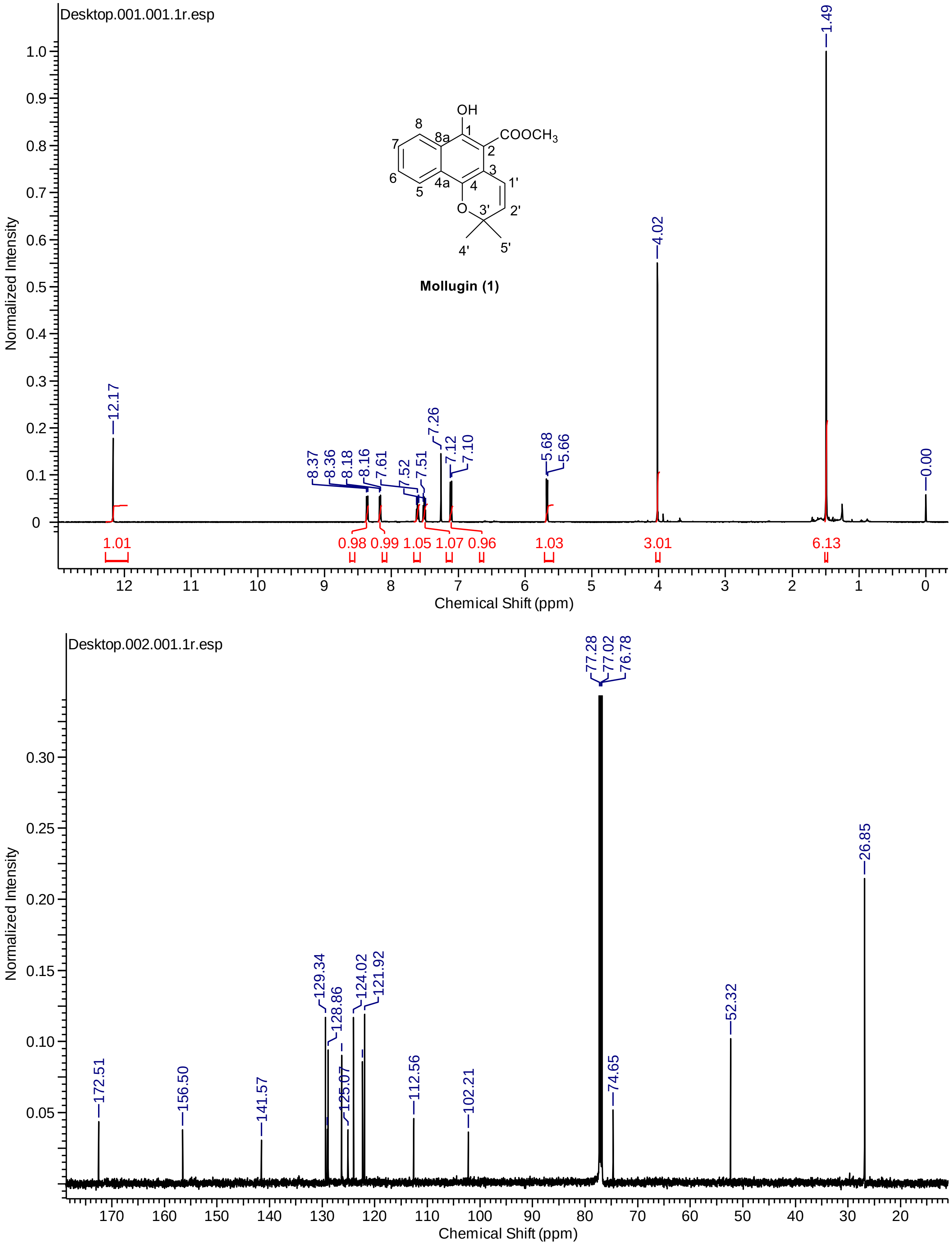

Figure S1. the representative ^{1}H, ^{13}C NMR spectra of mullugin isolated by CCC with the two-phase solvents of *n*-hexane-methanol-wate
